# 

**Published:** 1868-11

**Authors:** 


### NOTICE TO SUBSCRIBERS.

140 FULTON ST., N.Y., NOV. 1, 1868.

The editor of this Journal who has for several years been both Editor and Publisher, has at length made an arrangement by which he will in future be relieved from all the duties connected with the publication, with the single exception of those of the Editorial department. In this he is at home; in the publishing department he has simply been an interloper, exercising the prerogatives of a profession, for which, —as he is neither printer nor publisher—he has no diploma. For all the short-comings of the Journal in this respect, to the close of the present volume, he craves the indulgence of the subscribers. In future, this department will be confided to Mr. J. S. REDFIELD, 140 Fulton Street, whose reputation as an intelligent and tasteful publisher, is so well known as to need no endorsement. Relieved from this care, the Editor's attention will be directed solely, hereafter, to the improvement of the department under his especial charge.

W. W. HALL.

---

With the January (1869) number, the undersigned will commence the publication of a NEW SERIES of this Journal. Dr. Hall will continue to edit it, and being relieved from the duties of the business department, will be enabled to bestow more attention upon the preparation of matter for its columns.

This Journal will hereafter be printed and published in a style, not inferior to that of any other Magazine in the country. It will contain, as heretofore, twenty-four pages of reading matter in each number, and be published at one dollar and fifty cents per annum, *payable invariably in advance.*

Single numbers, twelve cents; if sent by Mail, prepaid, fifteen cents.

This Journal being stereotyped, bound volumes, and back numbers, from the commencement, can be supplied at any time.

☞ All communications relating to the business department of Hall's Journal of Health, should be addressed to

J. S. REDFIELD,

140 Fulton Street, N. Y.

